# The direct conversion of strictosamide to pumiloside *in planta* expands the camptothecin biosynthetic pathway

**DOI:** 10.1016/j.synbio.2026.05.006

**Published:** 2026-06-02

**Authors:** Yongpeng Li, Zhihan Wu, Junyu Chen, Qingyan Ruan, Lingtiao Yao, Lili Shao, Xiaolong Hao, Guoyin Kai

**Affiliations:** Zhejiang Provincial TCM Key Laboratory of Chinese Medicine Resource Innovation and Transformation, Zhejiang International Science and Technology Cooperation Base for Active Ingredients of Medicinal and Edible Plants and Health, Jinhua Academy, School of Pharmaceutical Sciences, Academy of Chinese Medical Sciences, Zhejiang Chinese Medical University, Hangzhou, 310053, China

**Keywords:** Camptothecin, Biosynthetic pathway, Spontaneous conversion, Medicinal plant, Synthetic biology

## Abstract

Cancer is a globally devastating disease that severely threatens human health and impedes social development. Camptothecin and its derivatives serve as crucial chemotherapeutic agents in clinical cancer treatment, owing to their unique inhibitory activity against DNA topoisomerase I (TOP1). Although the post-modifications of camptothecin (e.g., hydroxylation and subsequent methoxylation) have been well elucidated, the core biosynthetic pathway of camptothecin remains largely unclear. In the present study, we report an unexpected and significant finding: strictosamide can be directly converted to pumiloside both in several plant species (*Nicotiana benthamiana, Salvia miltiorrhiza*, and *Atractylodes macrocephala*) and in vitro. We term this process the “Direct Express Train” of the camptothecin biosynthetic pathway. We further demonstrate that this direct conversion proceeds more efficiently under alkaline conditions in vitro. Intriguingly, light was found to effectively facilitate this conversion. Under light exposure, exogenous flavin adenine dinucleotide (FAD) supplementation markedly promoted the reaction, with the final conversion rate reaching 38.8%. These findings not only deepen our understanding of the camptothecin biosynthetic pathway but also provide a novel strategy for the efficient biosynthesis of pumiloside (**7**) using plant chassis such as *N. benthamiana*.

Cancer remains one of the most devastating diseases globally, exerting a profound impact on human health and social development [1]. Camptothecin and its derivatives represent important chemotherapeutic drugs in clinical cancer therapy due to their unique DNA topoisomerase I (TOP1) inhibition activity. Camptothecin was initially isolated from *Camptotheca acuminata* and subsequently identified from diverse plant species such as *Nothapodytes nimmoniana*, *N. pittosporoides*, *Ervatamia heyneana*, and the herb *Ophiorrhiza pumila* [[2], [3], [4]]. Although the post-modifications of camptothecin, such as hydroxylation and subsequent methoxylation, have been well elucidated, the core biosynthetic pathway of camptothecin remains largely unknown [2,5,6]. It is proposed that the general MIA precursors strictosidine (**1**) (in *O. pumila*) and strictosidinic acid (in *C. acuminata*) form strictosamide (**2**) *via* a lactamization reaction [7,8]. Then, strictosamide (**2**) is converted to pumiloside (**7**) through five sequential catalytic steps, yielding four intermediates: strictosamide epoxide (**3**), strictosamide diol (**4**), strictosamide ketolactam (**5**), and 2-hydroxypumiloside (**6**) (Fig. 1A). The recent identification of strictosamide epoxide synthase and epoxide hydrolase (EH), which catalyze the formation of intermediates **3** and **4** from **2** [[3], [4], [5], [6]], respectively, has significantly advanced our understanding of the camptothecin biosynthetic pathway. Excitingly, herein we found that **2** can be directly converted to **7** in several plant species including *Nicotiana benthamiana*, *Salvia miltiorrhiza*, and *Atractylodes macrocephala*. In addition, we demonstrated that the conversion of **2** to **7** proceeded more readily *in vitro* under alkaline conditions. Intriguingly, we further confirmed that light could effectively facilitate this conversion. Under light conditions, exogenous supplementation of flavin adenine dinucleotide (FAD) markedly promoted the reaction, with the final conversion rate reaching 38.8%. These unexpected findings expanded our understanding on the camptothecin biosynthetic pathway, and led to the development of a novel method for the efficient biosynthesis of **7** in plant chassis such as *N. benthamiana.*

**Fig. 1:**
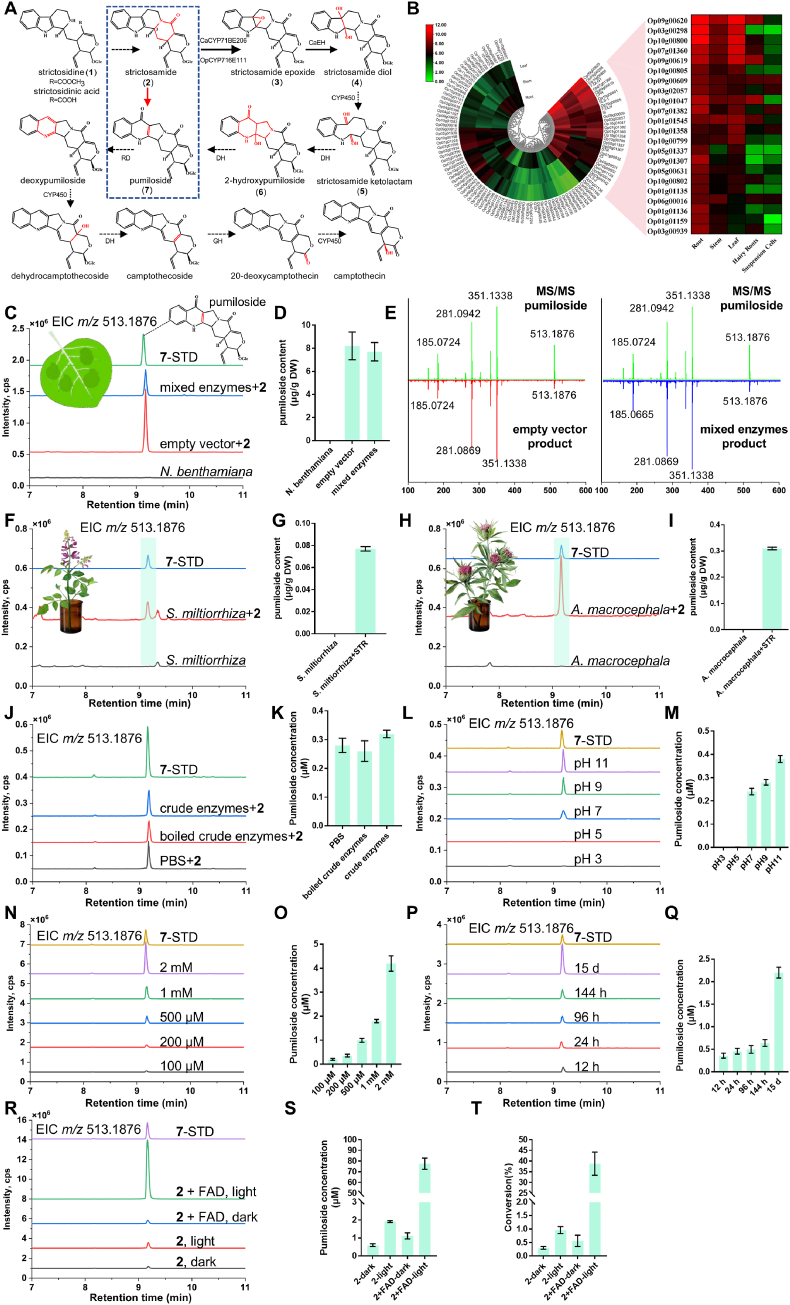
**The direct conversion of strictosamide to pumiloside in *planta* and *in vitro*. (A)** Proposed core camptothecin biosynthetic pathway. DH, dehydration. EH, epoxide hydrolase. GH, glycoside hydrolase, RD, reduction. **(B)** Co-expression analysis of *OpCYP450s* with the camptothecin biosynthetic pathway genes. The color scale indicates the Log2 (TPM values). **(C, D)** The direct conversion of strictosamide to pumiloside in the tobacco transient expression system. **(E)** The products showed an identical MS fragmentation pattern with pumiloside standard ([M+H]^+^: 513.1876, 351.1388, 281.0942, 185.0724). **(F, G)** The direct conversion of strictosamide to pumiloside in *Salvia miltiorrhiza.***(H, I)** The direct conversion of strictosamide to pumiloside in *Atractylodes macrocephala*. **(J, K)** The nonenzymatic conversion of strictosamide to pumiloside *in vitro*. **(L, M)** The conversion of strictosamide to pumiloside *in vitro* was associated with pH. **(N, O)** Yield of pumiloside under different concentrations of strictosamide. **(P, Q)** The conversion of strictosamide to pumiloside under various incubation durations. **(R, S)** The conversion of strictosamide to pumiloside under dark and light conditions in the presence or absence of FAD. **(T)** Conversion rates of strictosamide to pumiloside under dark and light conditions in the presence or absence of FAD. Data values are presented as mean ± standard deviation (n = 3) of three replicates.

It is well established that the two key intermediates **2** and **7** of the camptothecin biosynthetic pathway are positively correlated in *C. acuminata* [7]. However, the four intermediates **3**, **4**, **5** and **6** are present at extremely low levels in all *C. acuminata* tissues. Although the enzymes catalyzing the conversion of **2** to **4** have been identified, it remains a mystery whether a "Direct Express Train" from **2** to **7** exists. Given the advantage of high-quality genome and well-established genetic transformation system, *O*. *pumila* has emerged as an ideal model plant for elucidating the camptothecin biosynthetic pathway and its transcriptional regulatory mechanisms. Based on the co-expression analysis, a total of 22 CYP450 encoding genes were identified to be correlated with the camptothecin biosynthetic pathway genes such as *Op7-DLGT*, *OpCPR*, *OpSLS*, *Op7-DLH*, *OpIS*, *OpIO*, and *OpSTR* (Fig. 1B). Among these, 10 *CYP450* genes were associated with either the phenylpropanoid biosynthetic pathway or the upstream segment of the camptothecin biosynthetic pathway (Table S1). The remaining 12 *CYP450* genes were cloned into the plant expression vector *pHB* for transient expression in tobacco. Interestingly, when **2** was infiltrated into tobacco leaves, compound **7** was observed at *m/z* 513.1876 regardless of the presence of the mixed *Agrobacterium tumefaciens* harboring *pHB-CYP450* constructs at 3 days post infiltration (Fig. 1C–E). The resulting pumiloside contents were 7.7 μg and 8.2 μg (DW, dry weight), respectively (Fig. 1D). Considering that **2**, which contains an indole moiety, may be unstable under light [9], we performed a feeding assay using two additional medicinal plants in our laboratory, with compound **2** maintained in the dark. After a three-day feeding treatment in MM solution (200 μM **2**, 10 mM MgCl_2_, 10 mM MES, pH5.6), compound **7** was detected in *S. miltiorrhiza*, with a pumiloside content of 0.078 μg/g (DW) (Fig. 1F and G). Similarly, **2** was converted into **7** in *A. macrocephala*, yielding a pumiloside content of 0.3 μg/g (DW) (Fig. 1H and I)*.* These results suggested that **2** could be directly converted to **7** *in planta*.

To identify enzymes involved in this process in tobacco, crude enzyme extracts were prepared and subjected to catalytic assays. Reactions were performed with 200 ng of crude enzymes and 200 μM **2** in PBS buffer (pH7) supplemented with 1 mM NADPH, at 30 °C and 220 rpm for 12 h in the dark. Unexpectedly, comparable levels of **7** were also detected in the control groups lacking crude enzymes or containing boiled crude enzymes (Fig. 1J and K). Given that compound **2** was relatively stable in MM solution at pH5.6, we deduced that the conversion of **2** to **7** was associated with pH. As shown in Fig. 1L, M compound **7** was highly accumulated when the pH of the reaction buffer was adjusted to 7. The yield of **7** at pH7 was increased by 75.8-fold relative to that at pH5. In addition, we investigated the production of **7** at various substrate concentrations (100 μM, 200 μM, 500 μM, 1 mM, and 2 mM) after 12 h of reaction. The resulting **7** reached 4.2 μM at a compound **2** concentration of 2 mM (Fig. 1N and O). Furthermore, a time-course experiment was conducted using 200 μM **2** over a period spanning 12 h, 24 h, 96 h, 144 h, and 15 d. The yield of **7** exhibited a 6.1-fold increase by day 15 compared with the 12 h sample, reaching 2.2 μM (Fig. 1P and Q). Nevertheless, the *in vitro* conversion rates of these reactions remained low, ranging from 0.12% to 1.1%.

In addition, light was found to facilitate this conversion, with a corresponding rate of 0.96% (Fig. 1R–T). Previous study reported flavin-catalyzed oxidative indole ring opening [10]. Here, we found that under light conditions, exogenous FAD supplementation significantly accelerated the reaction, resulting in a final conversion rate of 38.8% (Fig. 1T). A plausible mechanism is that strictosamide acts as a photosensitizer to absorb light and generate singlet oxygen, while FAD functions as a cofactor to accept hydrogen atoms and electrons, thereby facilitating the sustained synthesis of pumiloside.

Overall, in this study, we unexpectedly discovered a "Direct Express Train" pathway from **2** to **7** both *in planta* and *in vitro*. Our results demonstrated that this direct conversion was markedly more efficient under alkaline and light conditions, with FAD serving as a key promoter that enhanced the photocatalytic conversion. These findings update the current model of the camptothecin biosynthetic pathway and provide a new strategy for the efficient biosynthesis of **7** using synthetic biology in plant chassis.

Detailed materials and experimental procedures are described in the Supplementary Information.

## CRediT authorship contribution statement

**Yongpeng Li:** Writing – review & editing, Writing – original draft, Visualization, Validation, Investigation, Funding acquisition, Conceptualization. **Zhihan Wu:** Writing – review & editing, Validation, Software, Investigation. **Junyu Chen:** Writing – review & editing, Investigation. **Qingyan Ruan:** Writing – review & editing, Investigation. **Lingtiao Yao:** Writing – review & editing, Investigation. **Lili Shao:** Writing – review & editing, Investigation. **Xiaolong Hao:** Writing – review & editing, Funding acquisition, Conceptualization. **Guoyin Kai:** Writing – review & editing, Supervision, Funding acquisition, Conceptualization.

## Declaration of competing interest

The authors declare that they have no known competing financial interests or personal relationships that could have appeared to influence the work reported in this paper.

## Data Availability

Data are available upon request to the corresponding author.
