# Supplementary material for: The direct conversion of strictosamide to pumiloside in planta expands the camptothecin biosynthetic pathway
Source: Synth Syst Biotechnol. 2026 Jun 2;14:413–6. doi: 10.1016/j.synbio.2026.05.006 (PMC13253211; doi:10.1016/j.synbio.2026.05.006)
Supplement: Multimedia component 1 [file mmc1.doc]

**SUPPLEMENTARY INFORMATION FOR**

**The direct conversion of strictosamide to pumiloside *in planta* expands the camptothecin biosynthetic pathway**

Yongpeng Li#, Zhihan Wu#, Junyu Chen, Qinyan Ruan, Lingtiao Yao, Lili Shao, Xiaolong Hao*, Guoyin Kai*

Zhejiang Provincial TCM Key Laboratory of Chinese Medicine Resource Innovation and Transformation, Zhejiang International Science and Technology Cooperation Base for Active Ingredients of Medicinal and Edible Plants and Health, Jinhua Academy, School of Pharmaceutical Sciences, Academy of Chinese Medical Sciences, Zhejiang Chinese Medical University, Hangzhou 310053, China

# These authors contributed equally to this work

* Correspondence author:

Guoyin Kai: [guoyinkai1@126.com](mailto:guoyinkai1@126.com)

Xiaolong Hao: [haoxiaolong@zcmu.edu.cn](mailto:haoxiaolong@zcmu.edu.cn)

**Materials and methods**

**Plant materials and chemicals**

Two-month-old *Nicotiana benthamiana*, *Salvia miltiorrhiza*, and *Atractylodes macrocephala* plants kept at Zhejiang Chinese Medical University (Hangzhou, China) were used for transient expression or feeding assays. All the plant materials were grown in a greenhouse at 25°C under a 16 h light photoperiod. The authentic standards strictosamide (**2**) and pumiloside (**7**) were purchased from PUSH BIO-TECHNOLOGY Co., Ltd. (Chengdu, China).

**Identification and cloning of candidate CYP450s**

Based on our previous study (Hao et al., 2023), all the identified CYP450s were used to build a heatmap with the camptothecin biosynthetic pathway genes. Total RNA was isolated using an OmniPlant RNA Kit (DNase I; Cowin Biotech, Taizhou, China). First-strand cDNA was synthesized using PrimeScript™ RT Master Mix (Takara, Shiga, Japan). Full-length cDNA sequences of *OpCYP450s* (*Op09g00609*, *Op03g02057*, *Op10g01047*, *Op07g01382*, *Op01g01545*, *Op10g01358*, *Op05g01337*, *Op10g00802*, *Op01g01135*, *Op06g00016*, *Op01g01136*, *Op01g01159*) were amplified with 2× KeyPo Master Mix (Dye Plus; Vazyme, Nanjing, China) on an Applied Biosystems™ 2720 Thermal Cycler. All primers used in this study are listed in Table S2.

**Transient expression assays in *N. benthamiana***

Transient expression assays were carried out as previously described. Briefly, the open reading frames (ORFs) of *OpCYPs* were ligated into *pHB-YFP* vector using *Bam*H I and *Spe* I restriction sites, in which the corresponding encoded proteins were fused with a yellow fluorescent protein (YFP). Then the resulting *pHB-OpCYP-YFP* plasmids were separately introduced into *A. tumefaciens* GV3101 and mixed for transient expression in *N. benthamiana*. After 3 days of injection, 200 μM strictosamide (**2**) dissolved in MM solution (10 mM MgCl2, 10 mM MES, pH5.6) were injected in to the leave infiltrated with the mixed *A. tumefaciens* strains. After another 3 days, the leaves were harvested and freeze-dried for metabolite extraction. All primers used here are listed in Table S2.

**Plant feeding assays**

*Salvia miltiorrhiza* and *Atractylodes macrocephala* plants were excised at the root and incubated in 200 μM strictosamide (**2**) dissolved in MM solution, with the treatment solution maintained in darkness. After 3 days of incubation, the leaves were harvested and freeze-dried for metabolite extraction.

**Metabolite extraction and UPLC-Q-TOF/MS analysis**

Dried leaves were ground into powder by high-speed bead beating at 55 Hz for 60 s in a TissueLyser. Then, 0.1 g of the dried leaf powder was extracted with 1 mL methanol in an ultrasonic bath (55 W, 1 h). The extracts were centrifuged for 10 min, and the supernatants were filtered through a 0.22 μm nitrocellulose membrane filter. Each extract (2 μL) was injected and analyzed using UPLC-Q-TOF/MS (Waters Xevo G2-XS). Chromatographic separation was performed on a Waters ACQUITY UPLC HSS T3 C18 column (100 mm × 2.1 mm, 1.8 μm) maintained at 30 °C. The mobile phase was composed of (A) 0.1% formic acid in water and (B) acetonitrile at a flow rate of 0.3 mL/min. The gradient elution program was set as follows: 0–15 min, 5% B; 15–20 min, 35% B; 20–25 min, 60% B; 25–30 min, 70% B; 30–32 min, 95% B; 32–35 min, 5% B. Mass spectrometry was operated using an electrospray ionization (ESI) source at 550 °C. The ion spray voltage was set to 5500 V in positive mode, with a curtain gas pressure of 35 psi. Multiple reaction monitoring (MRM) transitions were optimized with specific declustering potentials and collision energies for each ion pair. Mass scanning ranges from 50 to 1200 amu.

**Crude enzyme extraction and conversion of 2 to 7 *in virto***

*N. Benthamiana* leaves were used for the crude enzyme extraction as previously described (Huang et al., 2024; Guo et al., 2025). The extracted crude protein was combined, desalted and concentrated by using an Amicon Ultra centrifugal filter MWCO 30 kDa (Merck Millipore, Darmstadt, Germany) with PBS buffer (20 mM, pH7) at 7000 g at 4℃ for use in subsequent catalytic assays.

Enzymatic activity experiment was conducted in a 200 μL reaction buffer system containing 200 ng of purified crude enzymes and 200 μM **2** in phosphate buffer saline (PBS) (pH 7.0, without NaCl and KCl) supplemented with 1 mM NADPH. Reactions were incubated at 30 °C with shaking at 220 rpm for 12 h in the dark. The reaction was terminated by the addition of 200 μL methanol and mixed thoroughly. After a centrifugation at 12000 r/min for 10 minutes, the supernatant was filtered through a 0.22 µm organic filter membrane, and then detected by UPLC-Q-TOF/MS.

To determine the effect of pH, assays were performed with 200 μM substrate **2** in the following 20 mM buffers: citric acid-sodium citrate buffer (pH3), PBS (pH5, 7, 9), and sodium carbonate-bicarbonate buffer (pH11). Reactions were performed in 200 μL of sodium carbonate-bicarbonate buffer (pH11) with varying concentrations of substrate 2 (100 μM, 200 μM, 500 μM, 1 mM, and 2 mM) . All reactions were incubated at 30 °C with shaking at 220 rpm in the dark for 12 h. For the time-course assay, 200 μM of substrate 2 was incubated for 12 h, 24 h, 96 h, 144 h, and 15 d. Then, 200 μL of methanol was added to the reaction mixture and mixed thoroughly. After centrifugation at 12,000 rpm for 10 min, the supernatant was filtered through a 0.22 μm organic filter membrane and analyzed by UPLC-Q-TOF/MS.

For photocatalytic conversion assays, reactions were performed in 200 μL sodium carbonate-bicarbonate buffer (pH11) supplemented with 200 μM substrate 2, in either the presence or absence of 500 μM FAD, using LED lamps (Mulinsen MT8W96-30W; 3874.3 lx) as the light source.

**References**

Guo Z, Zhou Y, Li J, Liu D, Huang Y, Zhang Y, Yu R, Zhu J. Dihydroartemisinic acid dehydrogenase-mediated alternative route for artemisinin biosynthesis. Nat Commun. 2025, 16(1): 3888.

Hao X, Wang C, Zhou W, Ruan Q, Xie C, Yang Y, Xiao C, Cai Y, Wang J, Wang Y, Zhang X, Maoz I, Kai G. OpNAC1 transcription factor regulates the biosynthesis of the anticancer drug camptothecin by targeting loganic acid O-methyltransferase in *Ophiorrhiza pumila*. J Integr Plant Biol. 2023, 65(1): 133-149.

Huang Y, Wang H, Zhang Y, Zhang P, Xiang Y, Zhang Y, Fu R. SCPL acyltransferases catalyze the metabolism of chlorogenic acid during purple coneflower seed germination. New Phytol. 2024, 243(1): 229-239.
